# Cost and Data Quality Impact of Fraud Mitigation in Online Research: Framework Application and Evaluation Study

**DOI:** 10.2196/93951

**Published:** 2026-10-08

**Authors:** Maya Stemmer, Benjamin Buck, Justin Tauscher, Angelina Pei-Tzu Tsai, Anna Larsen, Patrick Wedgeworth, Gillian Sparks, Ella DeVries, Mercedes Bishop, Alexa Beaulieu, Trevor Cohen, Dror Ben-Zeev

**Affiliations:** 1 Behavioral Research in Technology and Engineering Center Department of Psychiatry and Behavioral Sciences University of Washington Seattle, WA United States; 2 Department of Psychiatry University of North Carolina at Chapel Hill Chapel Hill, NC United States; 3 Department of Biomedical Informatics and Medical Education University of Washington Seattle, WA United States

**Keywords:** CATCH framework, configure, assess, triage, corroborate, and hone, cost analysis, fraud mitigation, mental health, online research

## Abstract

**Background:**

Fully remote online health research is vulnerable to fraudulent participation that can threaten data validity and divert study resources. Although fraud mitigation is increasingly necessary in online research, mitigation efforts are rarely described systematically or evaluated transparently across studies.

**Objective:**

This study demonstrates the application of the configure, assess, triage, corroborate, and hone (CATCH) framework to an ongoing remote mental health study and evaluates how structured mitigation procedures have affected participant progression and operational costs.

**Methods:**

This study was conducted within an ongoing, fully remote mental health study evaluating hallucinations through longitudinal digital data collection. Following detection of fraudulent activity, the CATCH framework was applied to organize mitigation procedures into stages and refine detection and verification workflows. New screening safeguards were configured, classification thresholds were recalibrated, enrolled participants were reassessed and triaged accordingly, and secondary corroboration through synchronous video verification was introduced when warranted. Procedures were honed through ongoing monitoring in response to emerging fraud patterns. We compared 2 temporally defined cohorts before (March-August 2025) and after (September 2025-January 2026) the implementation of the refined procedures. Primary outcomes included the stage at which fraudulent participants were identified and cost per eligible participant, decomposed into costs attributable to fraudulent participants, nonfraudulent participants, and fraud-mitigation technology. Participant-level costs were estimated using a stage-based model incorporating participant compensation and research staff effort. Sensitivity analyses examined alternative compensation and staffing scenarios to assess the generalizability of the proposed cost-accounting mechanism.

**Results:**

Following CATCH implementation, fraudulent participants were identified earlier in the study workflow, reducing progression to later study stages. Overall, 41.5% (161/388) of reassessed participants were discharged for suspected fraud, while 58.5% (227/388) were retained following adjudication. Among those flagged as high risk and invited to video verification, 1.8% (3/164) successfully confirmed their identity, providing a lower-bound estimate of the false-positive classification rate. The mean sunk cost per fraudulent participant decreased from US $12.75 (SD US $40.76) in the pre-CATCH cohort to US $3.29 (SD US $10.91) in the post-CATCH cohort. Total cost per eligible participant decreased from US $170.48 to US $134.90, driven by a substantial reduction in the fraud-related portion from US $80.46 to US $19.73. Sensitivity analyses showed reductions in fraud-related cost per eligible participant across all alternative costing scenarios. To our knowledge, this is the first empirical demonstration of a structured fraud-mitigation framework implemented and evaluated within an active health study.

**Conclusions:**

Structuring fraud mitigation using an explicit framework enabled systematic documentation of procedural adaptations and their operational consequences. Beyond study-specific findings, this work illustrates how organizing fraud mitigation into defined, reportable stages can promote transparency, reproducibility, and cumulative learning across remote health studies. Treating fraud mitigation as an integral methodological component is essential for safeguarding scientific integrity and legitimate resource expenditure in online research.

## Introduction

Due to their scalability and reach, digital and fully remote approaches have grown more common in behavioral research. Alongside these advantages comes at least one persistent challenge: fraudulent participation [1,2]. Online recruitment with remote compensation is particularly vulnerable to identity falsification, duplicate enrollment, and the use of synthetic data [1,3-5]—behaviors that can distort recruitment sampling, compromise the validity of findings, and inflate study costs [6,7]. When fraudulent records remain undetected and progress through study procedures, they may contaminate analytic datasets and potentially alter study estimates and conclusions [8,9].

While several studies have shared examples of individual tools and strategies used to detect and manage fraud in online research [10-13], few have synthesized such tools into an integrated framework that allows for standardized reporting and shared learning across studies [9,14]. As fraudulent participation poses a systemic threat to the validity of health research, recent calls for action have emphasized the need for standardized, reproducible frameworks to guide and evaluate mitigation efforts [15,16].

The configure, assess, triage, corroborate, and hone (CATCH) framework [17] provides a roadmap for anticipating, detecting, and responding to fraudulent participation in online studies. It aims to mitigate fraud by balancing data integrity objectives with operational feasibility. CATCH begins with a prestudy *configuration* to prepare for fraud mitigation and establish safeguards and protocols. Once the study is underway, the *assess* stage involves systematically examining incoming data for irregularities or potential fraud indicators. Based on the assessment outcomes, candidates are *triaged* into risk categories, and those requiring further scrutiny move into the *corroborate* stage, where additional evidence is gathered to confirm or rule out fraudulent participation. The final *hone* stage outlines ongoing monitoring, evaluation, and refinement of mitigation procedures based on empirical outcomes.

This paper demonstrates the application of CATCH to an ongoing large-scale remote mental health study evaluating hallucinations and related outcomes through longitudinal digital data collection. To ensure the study produces valid and representative findings, our team is dedicating considerable human and technological resources to identify and expel fraudulent participants. Multiple safeguards were in place at study launch, but the scale and sophistication of fraudulent activity exceeded our expectations, prompting the development and implementation of more robust detection and verification procedures. CATCH provides a coherent framework for describing these adaptations and evaluating their impact on the course and cost of fraud mitigation. To quantify the operational burden in a manner adaptable across study designs, we developed a stage-based cost-accounting approach that tracks where and on whom study resources are expended, enabling transparent evaluation of the operational consequences of fraud mitigation decisions.

Accordingly, this study aimed to: (1) demonstrate the application of the CATCH framework within an ongoing online study, including how mitigation procedures were adapted as fraud patterns emerged; (2) examine how these procedural refinements affected fraudulent participant progression through study milestones; and (3) use the proposed cost-accounting approach to evaluate how procedural refinements affected the allocation of study resources, including costs attributable to fraudulent participation.

## Methods

### Ethical Considerations

The parent study was approved by the institutional review board of the University of Washington (ID STUDY00019781). All study participants provided electronic informed consent. The current study uses data collected under this approved protocol.

### Overview

The current manuscript analyzes the application of the CATCH framework within a large-scale, fully remote study developing data-driven clinical signatures for people who experience hallucinations. The parent study is described below to provide context regarding the recruitment, enrollment, and data-collection procedures within which fraud mitigation occurred. Fraud mitigation proceeded in 2 phases. Phase 1 involved strategies deployed from study launch until indicators of substantial fraud emerged 5 months later. In response, our team paused recruitment to refine mitigation and management procedures before resuming data collection. In phase 2, we developed and applied the CATCH framework. The configure, assess, triage, and corroborate stages outline the adaptation period, during which recruitment was paused. The hone stage describes the continued monitoring and refinement following the recruitment resumption and up to the writing of this manuscript. We evaluated the impact of these refinements by comparing fraudulent participant progression and study costs across temporally defined pre- and post-CATCH cohorts. Figure 1 provides a visual summary of the study workflow, including the 2 phases of fraud mitigation and the CATCH stages embedded within their corresponding periods.

**Figure 1:**
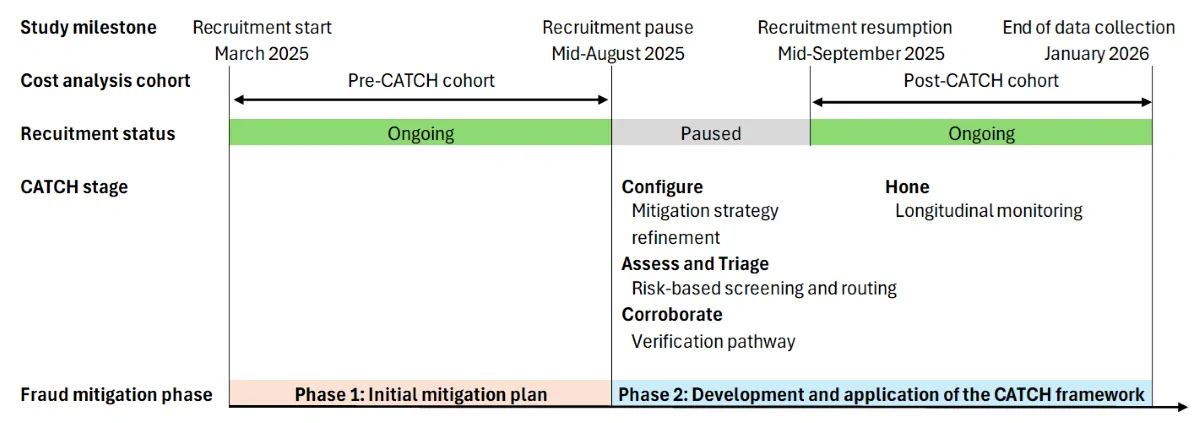
Study workflow and alignment with the configure, assess, triage, corroborate, and hone (CATCH) framework. The figure depicts the study course, recruitment status corresponding to the cost analysis cohort, and the 2 phases of fraud mitigation. The configure, assess, triage, and corroborate stages were applied during the recruitment pause, while ongoing monitoring and refinement (hone) began after recruitment resumed.

### Parent Remote Study: Developing Data-Driven Clinical Signatures for People Who Experience Hallucinations

#### Study Overview

The ongoing, fully remote observational study aims to enroll up to 2000 adults from the United States who experience hallucinations, with the goal of deriving data-driven “clinical signatures” that predict symptom course, functioning, and severe negative outcomes. Over the course of 12 months, participants are asked to complete a battery of online assessments as well as bursts of repeated measures using an integrated smartphone data collection system combining ecological momentary assessments (EMAs), cognitive tasks, and audio diaries describing their experiences of hallucinations. At the time of manuscript preparation, recruitment and follow-up assessments were ongoing; the procedures below describe the protocol as implemented.

#### Recruitment and Enrollment Procedures

Primary recruitment uses online outreach (social media and search advertisements), directing interested individuals to a study website with written information. A dedicated study email is available for candidates to contact with inquiries at any time. Interested individuals complete a web-based REDCap [18] screening questionnaire to assess eligibility. All study candidates complete an electronic informed consent form, which includes a brief quiz confirming their understanding of study procedures and objectives. Upon completion of consent, eligible participants are invited to complete the first assessment battery in REDCap. They later install the study mobile app on their personal smartphones, enabling subsequent mobile assessment.

#### Data Collection and Compensation

Data are collected at 6 time points throughout the study. Participants receive electronic compensation in the form of redeemable online gift cards, tied to study milestones. Specifically, participants receive US $50 for completing the first assessment battery, US $100 for completing a 1-month intensive follow-up, and US $30 for each completed 3-, 6-, 9-, and 12-month assessment. Research staff monitor participant engagement and data quality through administrative dashboards. Missed prompts trigger automated reminders, and staff conduct outreach to address technical issues or engagement barriers when needed.

### Phase 1: Initial Fraud Mitigation at Study Launch

#### Initial Safeguards and Screening Workflow

Participant recruitment began in March 2025. Screening procedures were initiated early in the recruitment period, and eligible candidates were enrolled on a rolling basis.

Multiple safeguards were incorporated into the study design to mitigate fraudulent participation during online recruitment. Prior to enrollment, candidates completed a CAPTCHA verification, structured attention checks, consistency checks, and an automated REDCap-based eligibility assessment. Candidates were required to provide identifying information (ie, name, email address, and phone number), and IP addresses were automatically collected. All information was manually reviewed by study staff to identify potential duplicates. To ensure comprehension and attention, candidates were required to pass a brief consent-knowledge questionnaire and answer attention checks throughout the screening procedure. CAPTCHA software and hidden-field items were included to detect automated or bot-generated responses. In addition, built-in REDCap checks automatically flagged inconsistencies between age and date-of-birth entries. The platform also captured system time zone data, which was compared against self-reported time zones to identify participants who reported a location different than that suggested by their device (as our team found to be common among fraudulent participants).

Based on the number and severity of flagged indicators, candidates were categorized into 3 fraud-risk tiers: low (green), medium (yellow), and high (red) risk. Individuals with no items flagged as suspicious were classified as low risk (green) and advanced to enrollment procedures. Those with 1-2 flagged indicators were classified as medium risk (yellow) and were subject to synchronous phone screening, wherein study staff further verified identifying and demographic details and assessed consistency in responses. Candidates with 3 or more flagged indicators were classified as high risk for fraudulent activity (red) and deemed ineligible to participate.

#### Emergence of Coordinated Fraud and Recruitment Pause

Study staff observed a substantial proportion of suspected fraudulent participants despite existing safeguards and routine monitoring. In response, the study made 3 adjustments to make screening more stringent. First, we added additional verification steps for current and future participants, comparing the consistency of self-reported zip codes and dates of birth across the screening and first assessment surveys. Second, we adjusted the thresholds defining the fraud-risk tiers so that candidates with 1 flagged indicator were classified as medium risk (yellow) and received a phone screen to determine eligibility, while those with 2 or more flagged indicators were classified as high risk (red) and automatically excluded.

Despite these more stringent standards, study staff continued to encounter odd or suspicious behavior from participants. Study staff witnessed a surge in participants asking to change their phone numbers and received repeated emails inquiring about payments. The requests, which arrived simultaneously from different participants, suggested duplicate and fraudulent enrollments.

In response, we evaluated the IP addresses of enrolled participants using an external fraud-detection service (IPQualityScore)—a method suggested in earlier work [6]. The service provides an IP risk score based on previous behavior logged for each IP (eg, a history of abusive or fraudulent activity, or the detection of a proxy or virtual private network). The test indicated that 42.3% (164/388) of active participants had IP addresses associated with “high risk” for fraudulent activity. The findings prompted a temporary suspension of recruitment between mid-August 2025 and mid-September 2025 to allow for a detailed evaluation of participants and study procedures. Our team’s response to this wave of fraud led to the development and application of the CATCH framework [17].

### Phase 2: Application of the CATCH Framework

#### Configure: Redesign of Safeguards and Operational Roles

The configure stage of CATCH refers to prestudy preparation, during which study-specific fraud assumptions, thresholds, and safeguards are defined before recruitment begins. Applying CATCH after the study commenced, we used this step to recalibrate the protocols and supervisory hierarchies that provide the structure for our fraud mitigation strategy. First, the mobile app was region-locked and restricted to the US app stores to reduce international traffic and limit the feasibility of repeated installation from nondomestic accounts. We also purchased a service (Blocky by Wix) to block non-US IP addresses from accessing the study landing page. Second, we added new checks to the screening workflow. We integrated the aforementioned IP risk-scoring tool into the screening of all entries. Low-risk IP scores did not require further follow-up, while high-risk IP scores warranted further investigation. We also introduced a source/link check to identify potential repeated enrollment. The study landing page used a different link to the initial REDCap screener for each recruitment wave, and candidates entering through an outdated or misaligned link were flagged as failing the source check. Third, study staff were instructed to screen out candidates whose activity was suspicious based on timing and surrounding context (eg, emails with a similar structure appearing in batches). As these entries were suggestive of coordinated efforts to defraud the study, these candidates were immediately deemed ineligible (red). Last, the study team added additional review steps based on patterns of suspicious behavior among some already enrolled participants. Study staff noticed a recurrent request to change the phone numbers used to enroll. As a response, when a participant requested a phone number change, staff members would check whether the new number was already in use in the longitudinal and screening databases. If duplicates were found, both the participant who requested the number change and the one with the matching number in the database were discharged from the study. If the new number was not a duplicate, the study staff would conduct additional phone screening at the new number provided.

All changes were systematized into standard operating procedures with detailed steps and responsibilities, escalation pathways, and decision thresholds for each fraud indicator. These configuration changes provided a foundation for more structured assessment and triage.

#### Assess: Systematic Identification of High-Risk Candidates

Following configuration of the revised fraud mitigation workflow, the team systematically applied the selected automated and manual checks at screening and throughout the study. The revised workflow combined safeguards already in place during phase 1 with the newly configured procedures described above. Incoming candidates were systematically screened using a predefined sequence of checks, intentionally ordered to prioritize low-cost, high-yield signals before applying resource-constrained review steps that required human effort.

In practice, the screening sequence began with time-zone verification for geographic validation, attention checks, and source/link checks. Candidates who passed initial screening were then evaluated using subsequent consistency checks, including cross-item response consistency and duplicate identifier checks. When no single check indicated a clear failure but multiple irregularities or atypical patterns were observed, research staff responded with additional adjudication. The IP risk score check, introduced during the configure stage, was applied after completing the above steps due to its reliance on an external service with usage limits. Outcomes of each check were recorded prospectively, and candidates were classified into predefined risk categories based on the cumulative results of the screening sequence, which informed subsequent triage actions.

#### Triage: Structured Allocation to Enrollment Pathways

Consistent with the triage stage of the CATCH framework, fraud risk assessment results were used to route candidates into predefined enrollment pathways. Candidates classified as low risk proceeded directly to the first assessment invitation and enrollment. Candidates classified as medium risk were routed to a mandatory staff-assisted phone screening, during which research staff verified key demographic details, discussed symptom history and lived experiences related to hallucinations, and administered additional rotating consistency checks on previously reported information, such as city of birth, highest level of education, and current time. These interactions provided an opportunity to identify inconsistencies or other indicators of potential eligibility misrepresentation before enrollment. Candidates classified as high risk were removed from the enrollment pipeline without further verification.

Following the introduction of IP-based risk scoring, previously enrolled participants whose IP scores exceeded the predefined threshold were also subject to triage decisions. Consistent with the risk-scoring algorithm, which incorporates indicators of spam activity, abusive behavior, and virtual private network or proxy use, scores above 50% were classified as high risk. Participants exceeding this threshold were therefore discharged from the study. Although this step was designed to prevent unnecessary compensation expenditures by enabling a mass drop of suspected fraudulent participants, it also raised the possibility of false exclusion (ie, excluding legitimate participants). To mitigate this risk, the study team created a verification pathway outside the primary enrollment flow, as described in the Corroborate: Secondary Verification to Confirm Eligibility section below.

#### Corroborate: Secondary Verification to Confirm Eligibility

##### Balancing Fraud Mitigation With False Exclusion

The corroborate stage of the CATCH framework addresses cases in which automated or early-stage screening procedures are insufficient to make final inclusion determinations. After the mass exclusion of participants identified as high risk through the updated assessment workflow, the study team recognized that some individuals may have been incorrectly classified due to IP-based risk inflation, shared networks, or data irregularities unrelated to fraudulent intent. To mitigate this risk, the team created a verification pathway outside the primary enrollment flow. This pathway provided participants with an opportunity to verify their identity synchronously.

##### Verification Procedures

Participants identified as having high-risk IP addresses were sent an email informing them that, due to suspicious activity, they were required to verify their identity in order to continue participation. Participants were asked to do so through a video call during which they presented one of several acceptable forms of documentation, such as a piece of mail showing their name and address, a state-issued identification card, or a passport. While allowing participants to submit a photo of their documentation instead of appearing on a live video call may have reduced participant burden, it would not have offered the same opportunity to verify participant information or clarify concerns in real time. The live interaction allowed research staff to confirm key demographic information and resolve inconsistencies that could not be evaluated through documentation alone.

Cases in which the individual was unable to provide documentation or could not verify their details resulted in a high-risk classification and removal from the study. Individuals who successfully completed the verification procedures were allowed to continue. All verification outcomes were documented, enabling the team to quantify the accuracy of high-risk classifications and the proportion of excluded participants later verified as legitimate.

#### Hone: Iterative Evaluation and Adaptation

##### Ongoing Monitoring and Procedural Refinement

Following the resumption of recruitment in mid-September 2025, fraud mitigation entered an ongoing refinement phase, consistent with the hone stage of the CATCH framework. Study procedures were continuously evaluated and prospectively updated in response to emerging patterns observed during active recruitment and longitudinal follow-up.

Several additional assessment checks were introduced after recruitment resumed to evaluate consistency across independently collected administrative and behavioral data sources. The study implemented a consistency check comparing IP-derived geographic information with self-reported state of residence. Cases in which the derived location did not align with self-reported geographic information were flagged for secondary phone screen verification. An additional geographic consistency rule was introduced to compare self-reported zip codes and time zones. As with the IP geographic check, misalignment between zip code and time zone fields triggered further review and routing to phone screening.

Verification and triage workflows were further refined to address emerging patterns in administrative and engagement-related processes. Requests to change participant phone numbers triggered mandatory screening calls to confirm identity and eligibility prior to updating contact information. Participants who did not respond to the call were given 1 week to contact study staff to complete a confirmation call. Failure to do so resulted in discharge from the study.

Throughout the hone phase, study staff continued to monitor consistency across first and follow-up assessments, engagement with EMAs, and adherence to verification procedures. One ongoing monitoring process involved comparing first-assessment responses with 1-month follow-up survey responses. Research staff reviewed responses for discrepancies in administrative data, including zip codes and dates of birth. If misalignment was identified between surveys, a phone screening was conducted during which participants were asked to confirm their zip code and date of birth. If inconsistencies persisted following verification, participants were removed from the study. Research staff also had the discretion to ask additional questions as needed to verify participant authenticity.

Audio diary submissions provided an additional source of corroboration during ongoing monitoring. The first several submissions from each participant were reviewed to identify duplicate recordings or recordings with insufficient audio quality and to assess whether participants’ responses were consistent with their reported demographic characteristics (eg, age, gender, and race), as well as the expected presentation of the target population.

Retention-related discharge criteria were also refined during this period. Updated rules were implemented to distinguish between acceptable nonresponse and prolonged absence of data submission across follow-up time points, with the goal of maintaining data integrity while minimizing premature exclusion. Participants who submitted no data via the study app or assessment batteries at both the 3- and 6-month follow-up time points were considered lost to follow-up.

The cumulative refinements to the study’s fraud mitigation procedures are summarized in Table 1, which provides a side-by-side comparison of the fraud mitigation safeguards implemented before and after application of the CATCH framework.

**Table 1:** Comparison of fraud mitigation safeguards implemented before (phase 1) and after (phase 2) application of the CATCH^a^ framework.

Safeguard domain		Strategy	Phase 1	Phase 2
**Applied before enrollment during screening**				
	Automated checks for bot detection	CAPTCHA verification and hidden-field items	Implemented	Implemented
	Automated consistency checks	Built-in REDCap checks comparing age and date-of-birth entries, and system-captured time zone with self-reported time zone	Implemented	Implemented
	Duplicate checks	Duplicate contact information (email address, phone number, and IP address)	Implemented	Implemented
	Fraud-risk tiers	Risk classification and corresponding action: low risk (enrolled), medium risk (phone screen), and high risk (not enrolled)	Low: 0 flags; medium: 1-2 flags; high: ≥3 flags	Low: 0 flags; medium: 1 flag; high: ≥2 flags
	Geographic restrictions	Restriction of app availability to US app stores	Not implemented	Implemented
	Geographic restrictions	Blocking access from non-US IP addresses	Not implemented	Implemented
	IP risk assessment	External IP risk-scoring service; high-risk scores triggered manual review	Not implemented	Implemented
	Manual review of suspicious submission patterns	Exclusion based on timing and surrounding context (eg, email structure and submission batches)	Not implemented	Implemented
	Recruitment source control	Separate recruitment link for each recruitment wave; outdated or misaligned links failed the source check	Not implemented	Implemented
**Applied after enrollment during study participation**				
	Audio submission review	Assessment of quality, duplicate submissions, consistency with reported demographics, and alignment with the expected presentation of the target population	Implemented	Implemented
	Longitudinal consistency checks	Consistency checks of self-reported zip code and date of birth across screening and first assessment	Not implemented	Implemented
	Phone change procedure	Phone screening the new number, after ensuring it isn't already in use in the study	Not implemented	Implemented
	Geographic consistency	IP-derived state vs self-reported state	Not implemented	Implemented
	Geographic consistency	Zip code vs time zone	Not implemented	Implemented

^a^CATCH: configure, assess, triage, corroborate, and hone.

##### Cost Analysis

To examine the impacts of applying CATCH to our study and evaluate the operational burden of fraudulent participation, we conducted a cost analysis comparing the 2 fraud-mitigation phases. Cost in remote studies accumulates as participants progress through screening and early follow-up activities. Following the mass drop of fraudulent participants, we refined procedures to detect fraudulent candidates earlier and reduce both staff workload and participant compensation. Comparing costs before and after these changes provides a pragmatic measure of the operational impact of the revised mitigation workflow.

The analysis incorporated four sources of cost directly tied to fraud mitigation: (1) participant compensation, based on the stage each participant reached (eg, first assessment and follow-up visits), with deeper progression resulting in higher compensation cost; (2) research staff time, including minutes spent on initial screening review, as well as enrollment and monitoring procedures, monetized using an average salary-per-minute estimate; (3) phone verification workload, captured as part of research staff time and indicating operational burden of inconclusive cases; and (4) fraud mitigation technology (ie, any paid software or tool implemented for fraud detection).

To estimate participant-level cost, we combined stage-specific compensation with research staff effort. For each participant, compensation reflected accumulated incentives, and staff time reflected the set of verification and manual review activities applied. For each participant *i*, the total cost was defined as:

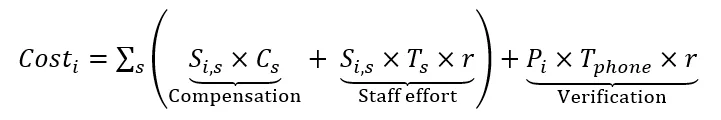



where *S_i,s_* = 1 if participant *i* reached stage *s*, 0 otherwise; *C_s_* is the compensation for stage *s*; *T_s_* is research staff time (in minutes) required for stage *s*; *r* is the staff rate per minute; and *P_i_* = 1 if participant *i* completed phone verification, 0 otherwise. Using equation 1, we calculated both participant-level cost and aggregated cost patterns across fraudulent and nonfraudulent participants (ie, those not identified as fraudulent), as well as compared pre- and post-CATCH cost distributions.

We compared participant-level costs across 2 temporally defined cohorts corresponding to periods before and after the mass drop of fraudulent participants. The pre-CATCH cohort included all candidates and participants who initiated screening prior to when recruitment was paused. For this cohort, participant progression, outreach activity, compensation, and research staff effort were censored at the pause date to reflect only costs accrued under the initial procedures. Adjudication of suspected fraudulent cases continued during the recruitment pause, and the final fraud classification for the pre-CATCH cohort reflects the information available to the study team during that period.

The post-CATCH cohort included only candidates and participants who initiated screening after recruitment resumed. For this cohort, a snapshot was taken after 4 months, and all participant progression, outreach, and compensation events occurring until that point were included. As a result, our analysis was based on 2 mutually exclusive cohorts of similar length, allowing compensation to accumulate up to the 3-month time point but not beyond.

To operationalize the participant-level cost model, we applied fixed, study-specific parameters that reflect both participant compensation and research staff effort. Time estimates (in minutes) were assigned to each study stage based on standard operating procedures: initial screening (2 minutes), enrollment processing (5 minutes), first assessment completion (2.5 minutes), follow-up visit review (1.5 minutes), and staff-assisted phone screening (5 minutes). Participant incentives were defined by protocol and held constant across cohorts, with compensation of US $50 for completing the first assessment, US $100 for the 1-month follow-up, and US $30 for subsequent follow-up visits. Consistent with staff salary rates, staff effort was valued at US $35 per hour for cost estimation purposes.

Time estimates for each study activity were obtained from the 2 research staff members who had conducted participant screening, verification, and enrollment procedures throughout the study. Estimates reflected the average time required for each activity based on approximately 1 year of operational experience with the protocol. Together with the staff hourly rate, these values were used to represent the resource requirements of the present study. Because the cost model is parameterized, investigators can substitute study-specific estimates to account for differences in staffing and study workflows.

Fraud mitigation technology costs were incorporated at the cohort level in addition to participant-level costs. The IP risk-scoring service, implemented using its free tier, incurred no direct cost. The IP-blocking service incurred a fixed monthly subscription fee of US $11.02, independent of usage volume, beginning in mid-August 2025. A fixed cost of US $66.12 was therefore added to the total cost of the post-CATCH cohort, reflecting 6 months of service from August 2025 through January 2026.

Costs were summarized separately for participants classified as fraudulent and nonfraudulent. Because eligibility was assessed before fraud screening, candidates who did not meet eligibility criteria did not proceed through the fraud adjudication process and were therefore classified as nonfraudulent for cost-accounting purposes. We compared distributions of total participant-level cost pre- and post-CATCH. Costs per eligible participant were calculated by dividing the total resources expended across all candidates and participants—including those later adjudicated as fraudulent or dropped for non-fraud-related reasons—and fixed fraud mitigation technology costs by the number of eligible participants. We decomposed this metric into costs attributable to fraudulent participants, nonfraudulent participants, and fraud mitigation technology.

To demonstrate a generalizable cost-accounting approach and illustrate how these findings may inform other studies, we re-estimated costs under alternative parameterizations [19,20] of 2 core dimensions: participant compensation structure and research staff effort. Scenarios were selected to reflect common design variations in online research, including studies with evenly distributed incentives across stages, studies that concentrate compensation at later milestones, and studies with substantially higher staff burden, such as qualitative interviews. The full parameter values for each scenario are provided in Multimedia Appendix 1.

## Results

### Application of the CATCH Framework

#### Configure, Assess, and Triage: Reclassification of Participants Under Updated Procedures

Prior to the revised screening definitions and workflow, 3562 study candidates were screened and classified. Of these, 821 (23%) were excluded for failing to meet eligibility criteria. Among the remaining 2741 candidates, 542 (19.8%) were classified as low risk, 387 (14.1%) as medium risk, and 1812 (66.1%) as high risk based on the combined automated and manual checks.

Following revised triage and workflow definitions, all enrolled participants who remained active at the time of reassessment were re-evaluated using the updated fraud indicators. A total of 388 participants were reviewed during this period. Of these, 30 (7.7%) had previously passed a phone screen and were retained without further verification. Among the remaining 358 participants, 194 (54.2%) had low-risk IP scores and were retained, while 164 (45.8%) had high-risk IP scores and were invited to complete the verification pathway.

#### Corroborate: Verification Pathway Participation

The flow of participants through the verification pathway is shown in Figure 2. Of the 164 participants deemed high risk based on their IP scores, 149 (90.9%) did not respond to the invitation to schedule a video call to confirm their identity and were discharged from the study after the grace period ended. Only 15 (9.1%) reached out to schedule a video call. Of this subgroup, 12 (7.3%) missed their scheduled verification meetings and were given the opportunity to reschedule; they were ultimately discharged for failing to attend or reschedule. The remaining 3 (1.8%) attended the video call meeting and successfully confirmed their identity.

**Figure 2:**
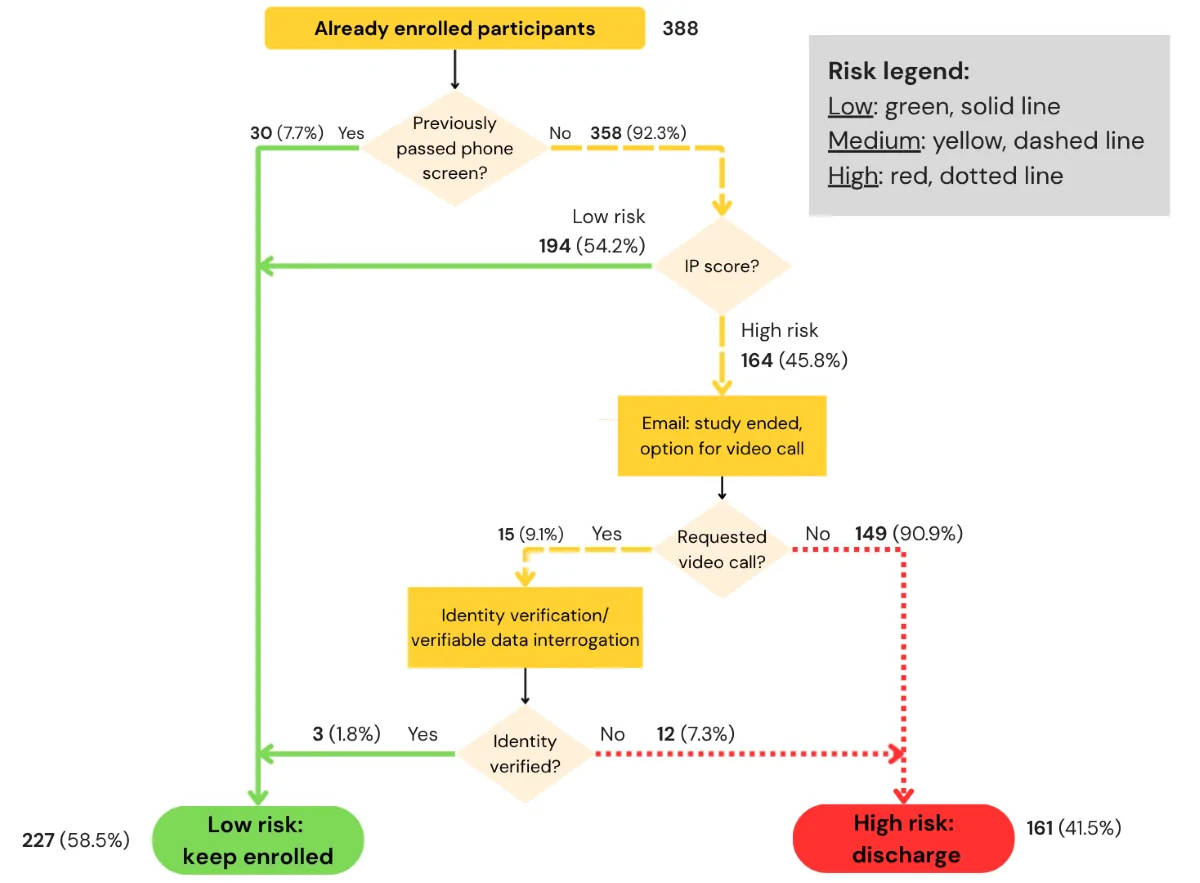
Enrolled participant flow chart, including IP risk score check results and verification pathway. Percentages are calculated relative to the population entering each decision pathway, with final outcomes calculated relative to all 388 reassessed participants.

In total, the mass drop of enrolled participants (n=388) based on their IP risk score resulted in 227 participants (58.5%) retained in the study and 161 (41.5%) discharged for unresolved suspicion associated with their high IP risk score. The lower bound of the proportion of false positives generated by the IP risk score workflow can be estimated at 1.8% (3/164)—those who passed video verification among those who were required to do so.

#### Hone: Ongoing Monitoring and Strategy Refinement

Following recruitment resumption, additional geographic consistency checks were introduced as part of ongoing monitoring. A comparison of IP-derived geographic location with self-reported state of residence identified 112 misaligned cases among new study candidates. Of these, 70 (62.5%) failed additional checks and were classified as high risk, leading to their removal from the study. The remaining 42 (37.5%) were classified as medium risk and routed to secondary phone screening; only 5 passed verification and were retained.

A second geographic consistency check comparing self-reported zip codes and time zones was conducted among 286 active participants at the time, identifying 3 (1%) suspected cases. These participants were routed to phone screening, and 1 passed verification.

During ongoing monitoring, 7 participants were removed due to persistent audio quality issues despite repeated outreach. Additional participants were removed following detection of duplicate audio submissions; however, duplicate audio recordings could not be reliably disaggregated from other forms of duplication (eg, reuse of phone numbers) in retrospective records.

### Cost Analysis

Across cohorts, participant-level costs differed substantially by fraud classification. The mean sunk cost of fraudulent records in the pre-CATCH cohort was US $12.75 (SD US $40.76), compared with US $3.29 (SD US $10.91) in the post-CATCH cohort, reflecting deeper study progression prior to detection. Under the refined procedures, fraudulent participants were more frequently identified earlier in the workflow, resulting in lower incentive payouts and reduced staff verification effort. Specifically, among the 2366 fraudulent participants in the pre-CATCH cohort, 2168 (91.6%) were identified at screening, 56 (2.4%) during the first assessment, 83 (3.5%) at the 1-month assessment, and 59 (2.5%) at the 3-month assessment. Among the 1452 fraudulent participants in the post-CATCH cohort, 1411 (97.2%) were identified at screening, 37 (2.5%) during the first assessment, 3 (0.2%) at the 1-month assessment, and 1 (0.1%) at the 3-month assessment.

Nonfraudulent records showed the opposite pattern. The mean total cost of nonfraudulent records in the post-CATCH cohort was US $61.65 (SD US $72.18), compared with US $28.23 (SD US $56.04) in the pre-CATCH cohort, reflecting greater progression through study milestones among nonfraudulent participants at the time of analysis. While final retention cannot yet be determined because the study is ongoing, this pattern suggests that refined procedures did not broadly suppress legitimate participation but instead shifted operational resources away from fraudulent participation and toward nonfraudulent candidates and participants.

Table 2 presents cohort-level cost accounting metrics before and after the procedural refinements. Whereas the participant-level estimates above describe the mean cost incurred per fraudulent or nonfraudulent record, the cohort-level estimates normalize total expenditures by the number of eligible participants obtained. Each cost component (total, fraudulent, or nonfraudulent) was divided by the number of eligible participants, representing the study resources invested to obtain 1 member of the intended study population.

**Table 2:** Observed study costs per eligible participant by cohort.

Study measure				Pre-CATCH^a^		Post-CATCH		Δ (post-CATCH – pre-CATCH)
**Screened, n**				3562		1903		–1659
	Fraudulent		2366		1452		–914	
	Nonfraudulent		1196		451		–745	
		Ineligible	821		209		–612	
		Eligible	375		242		–133	
**Total cost (US $)**				63,929.83		32,645.12		–31,284.71
	Fraudulent		30,172.46		4775.25		–25,397.21	
	Nonfraudulent		33,757.38		27,803.75		–5953.62	
	Fraud mitigation technology		0.00		66.12		66.12	
**Cost per eligible participant (US $)**				170.48		134.90		–35.58
	Fraudulent portion		80.46		19.73		–60.73	
	Nonfraudulent portion		90.02		114.89		24.87	
	Fraud mitigation technology portion		0.00		0.27		0.27	

^a^CATCH: configure, assess, triage, corroborate, and hone.

The total cost per eligible participant decreased following the procedural refinements (pre-CATCH: US $170.48 vs post-CATCH: US $134.90). Decomposing this difference revealed that fraud-related cost per eligible participant decreased substantially (pre-CATCH: US $80.46 vs post-CATCH: US $19.73), while the nonfraudulent cost per eligible participant increased (pre-CATCH: US $90.02 vs post-CATCH: US $114.89). Thus, the overall reduction in cost per eligible participant was driven by substantially lower fraud-related expenditures, while still preserving nonfraudulent participant progression. The specific cost values reported in Table 2 are study-dependent and reflect our study’s structure and payment mechanism. However, the analytic structure—modeling cost as a function of stage reached and verification effort—can be readily adapted to other study designs.

Table 3 presents the replication of our results across other study designs using different combinations of incentive and staff effort values to illustrate the generalizability of the findings. The evaluated scenarios represent common variations in online research, including evenly distributed incentives across study stages, end-loaded compensation schedules, and studies with substantially higher staff burden. The full parameter values for each scenario are provided in Multimedia Appendix 1.

**Table 3:** Replication and comparison of the total costs per eligible participant results across different study designs. Each column represents deltas between the post-CATCH^a^ and pre-CATCH cohorts.

Study measure		Observed study		Equal incentives		End-loaded compensation		High staff effort
**Total cost (US $)**		–31,284.71		–31,754.71		–25,154.71		–43,624.38
	Fraudulent	–25,397.21	–8683.62		–10,783.62		–11,346.25	
	Nonfraudulent	–5953.62	–23,137.21		–14,437.21		–32,344.25	
	Fraud mitigation technology	66.12	66.12		66.12		66.12	
**Cost per eligible participant (US $)**		–35.58		–41.42		–49.62		–58.18
	Fraudulent portion	–60.73	–54.50		–33.79		–77.83	
	Nonfraudulent portion	24.87	12.80		–16.10		19.38	
	Fraud mitigation technology portion	0.27	0.27		0.27		0.27	

^a^CATCH: configure, assess, triage, corroborate, and hone.

Across all alternative costing scenarios, procedural refinement reduced the total cost per eligible participant, with reductions ranging from US $35.58 under the observed study parameters to US $58.18 under the high staff-effort scenario. These reductions were driven by lower fraud-related costs, which decreased across all scenarios. Nonfraudulent costs increased under the observed study, equal-incentive, and high staff-effort scenarios, but decreased under end-loaded compensation. The magnitude of fraud-related cost reductions varied with study design assumptions, ranging from US $33.79 under end-loaded compensation to US $77.83 under the high staff-effort scenario. Overall, the sensitivity analysis indicates that the reduction in fraud-related costs per eligible participant was robust across the modeled scenarios, while its magnitude depended on where study costs accrued and how participant compensation was staged over time.

Overall, the hone phase demonstrated that ongoing monitoring and procedural refinement altered the timing and depth of fraudulent participant progression through the study. By detecting fraudulent activity earlier and reducing unnecessary downstream engagement, refined procedures decreased the costs associated with fraudulent participation while preserving investment in eligible participants.

## Discussion

### Principal Findings

In this empirical application of the CATCH framework to a large remote observational mental health study, we demonstrate how fraud mitigation procedures can be systematically organized, documented, and evaluated in real-world conditions. Following a recruitment pause prompted by emerging evidence of coordinated fraudulent participation, procedural refinements were introduced. New detection strategies were incorporated, and ongoing monitoring was sharpened, shifting fraud detection earlier in the study workflow and reducing downstream operational costs associated with fraudulent participation. CATCH scaffolded our approach; it provided a consistent way to delineate stages of fraud mitigation, document how procedures were deployed within each stage, and quantify the timing and operational implications of those procedures. To our knowledge, this is the first study to apply a structured fraud mitigation framework and to provide empirical evidence showing how such frameworks can be implemented and evaluated within active health studies.

The high levels of fraudulent participation observed in this study are not unique but reflect a broader challenge facing online health research. Recent work identified fraudulent participation among 62% of eligible screeners in a decentralized digital mental health trial, with additional fraudulent participants identified after enrollment [6]. A recent scoping review of online health research reported fraudulent participation rates ranging from 3% to 94%, depending on study design, recruitment methods, and detection procedures used [21]. The magnitude of the problem highlights the need not only for effective fraud mitigation but also for transparent frameworks to document and evaluate such procedures across studies.

### Fraud Detection Timing and Operational Impact

Fraudulent participants who advance through multiple stages drain project resources; they consume compensation funds, research staff time for screening and verification, and additional administrative effort. A central finding of this study is that procedural refinement was associated with earlier identification of fraudulent participation, as reflected by reduced progression of fraudulent cases into later study stages. Earlier exits from the workflow reduced both false participant incentive expenditures and staff effort spent on fraudulent cases, resulting in lower total costs. Costs in the post-CATCH cohort were driven primarily by deeper progression and follow-up among eligible participants, consistent with improved detection of fraudulent participation earlier in the study workflow.

In longitudinal online research, participant engagement often unfolds across multiple time points, and late-stage exclusion can carry substantial financial and logistical costs. The results underscore that the placement and sequencing of mitigation procedures, rather than their mere presence, can meaningfully influence both detection timing and operational burden. By quantifying cost accumulation as a function of stage reached, the analysis offers an interpretable and reproducible approach for evaluating the downstream impact of fraud mitigation strategies.

### Generalizability of the Cost-Accounting Approach

Although the specific cost values reported here are study-dependent and reflect this study’s incentive structure, staffing model, and follow-up schedule, the analytic structure underlying the cost analysis is portable. By decomposing cost into stage-specific participant compensation and research staff effort, the suggested mechanism can be adapted to a wide range of study designs. As further illustrated by our sensitivity analysis, alternative parameterizations of incentives and staff effort may shift the balance between fraud-related and eligible-participant costs. The included scenarios reflect common design choices in online research, such as staff-intensive qualitative interviews and end-loaded compensation, allowing investigators to reason about cost tradeoffs in relation to their own workflows. Across scenarios, refined procedures consistently reduced fraud-related sunk costs while preserving or enhancing eligible participant progression, reinforcing the value of stage-based cost monitoring as a tool for operational decision-making.

In our sensitivity analysis, end-loaded compensation showed the smallest reduction in fraud-related costs and was the only scenario to show a decrease in nonfraudulent costs. While fewer incentives were issued before fraudulent participants were excluded, compensation to nonfraudulent participants was also delayed. Recent studies have recommended tying compensation to participant adjudication, including suggestions to delay or condition compensation until participant eligibility and data quality can be verified [22] or to offer no compensation for baseline surveys in longitudinal studies [6]. While delaying compensation may reduce fraudulent participation, it may also create additional barriers for legitimate participants, particularly those from economically vulnerable or historically underrepresented populations [13]. Compensation structure should be considered not only as an incentive strategy but also as a design decision that influences both fraud vulnerability and participant inclusion.

### Implications for Online Research Practice

Fraud mitigation in online research is often addressed reactively, after data quality concerns become apparent. Our findings highlight the importance of treating fraud mitigation as an integral component of study design and monitoring, rather than an ad hoc response to emerging problems. As fraudulent participation becomes increasingly sophisticated, no single mitigation strategy will be sufficient across contexts. Adopting a shared structure for organizing, documenting, and evaluating mitigation efforts can promote transparency, comparability, and cumulative learning across studies.

By organizing fraud mitigation into explicit, reportable stages, the CATCH framework enables researchers to move beyond isolated narratives of fraud and toward systematic evaluation of mitigation strategies and their consequences. Such standardization allows research teams and reviewers alike to assess not only whether fraud was addressed, but how mitigation efforts evolved over time and at what operational cost.

### Limitations and Future Directions

Several limitations should be acknowledged. First, fraud adjudication in online research is imperfect. As in other online studies [9,23], fraud status in our analysis reflects the best available information at the time of data collection and review. It is possible that we inadvertently deemed legitimate participants fraudulent, or that some participants classified as legitimate remained undetected. Accordingly, all results should be interpreted as conditional on the study’s evolving fraud-detection procedures and knowledge at the time of analysis. As fraud-mitigation procedures may themselves affect who is willing or able to participate in online research, one possible direction for future research is to examine whether such procedures systematically affect sample composition and representativeness.

Second, the cost analysis presented here is intended to demonstrate a generalizable accounting structure rather than to estimate absolute or optimal costs. While we show how modeling cost accumulation by study stage can make the operational consequences of fraud mitigation decisions explicit, the specific parameter values used reflect our study context. The sensitivity analyses illustrate how different study designs may shift cost allocation but are not intended to predict cost outcomes in other settings. Moreover, our cost model incorporated fixed fraud-mitigation technology costs but did not account for usage-based technology costs. Future work should expand this framework to accommodate alternative pricing structures, such as per-use or volume-dependent costs, as paid fraud-detection tools become increasingly integrated into mitigation pipelines.

Fraud mitigation in online research is inherently adaptive. Investigators are unlikely to anticipate all forms of fraudulent participation a priori, and reliance solely on predefined safeguards may prove insufficient over time. The CATCH framework does not eliminate this uncertainty but provides a structure for monitoring where vulnerabilities emerge and for evaluating procedural responses as fraud patterns evolve. Future work should focus on prospective study designs that explicitly plan for such uncertainty by embedding mechanisms for ongoing evaluation and adaptation, rather than treating fraud mitigation as a one-time or reactive task. Such approaches may be particularly important in long-running or high-incentive studies, where fraudulent strategies are likely to evolve as the study progresses.

### Conclusion

Fraudulent participation in online research presents a threat to data integrity and drains project resources. By structuring mitigation efforts prospectively and evaluating their operational impact, researchers can better anticipate where vulnerabilities may arise and make informed decisions about how to mitigate fraud.

While no single mitigation strategy is sufficient for all contexts, a shared framework such as CATCH can promote transparency and support cumulative learning across studies. Researchers should plan for fraud mitigation early in study design while remaining prepared to adapt their methods as studies progress, technologies evolve, and new patterns of fraudulent behavior emerge. As online and decentralized studies continue to expand, treating fraud mitigation as an integral, evolving component of study design will be essential to safeguarding scientific integrity and legitimate resource expenditure.

## Data Availability

The datasets generated and analyzed during this study are not publicly available due to the sensitive nature of the data and the potential for participant identification, particularly given the inclusion of fraud detection indicators and administrative metadata. Deidentified and aggregated data supporting the findings may be available from the corresponding author upon reasonable request and subject to institutional approval.

## Appendix

Multimedia Appendix 1 Parameter values used in scenario-based sensitivity analyses.

## Abbreviations

CATCH: configure, assess, triage, corroborate, and hone
EMA: ecological momentary assessment
