# Supplementary material for: Cost and Data Quality Impact of Fraud Mitigation in Online Research: Framework Application and Evaluation Study
Source: J Med Internet Res. 2026 Oct 8;28:e93951. doi: 10.2196/93951 (PMC13691467; doi:10.2196/93951)
Supplement: Supplementary file 1 [file jmir_v28i1e93951_app1.doc]

**Supplementary Table 1. Parameter values used in scenario-based sensitivity analyses.**

Sensitivity analyses were conducted by re-running the cost-accounting model under alternative incentive schedules and staff-effort assumptions to reflect different study designs.

|  |  | **Incentives ($)** | | | **Staff effort (minutes)** | | | | |
| --- | --- | --- | --- | --- | --- | --- | --- | --- | --- |
| **Scenario** | **Scenario description** | **First assessment** | **1M follow-up** | **3M follow-up** | **Screening** | **Enrollment** | **First assessment** | **1M follow-up** | **3M follow-up** |
| Primary (Observed in current study) | Observed study incentives and staff effort assumptions used for main analyses | 50 | 100 | 30 | 2 | 5 | 2.5 | 1.5 | 1.5 |
| Scenario A: Equal incentives | Total incentive budget distributed equally across stages | 60 | 60 | 60 | 2 | 5 | 2.5 | 1.5 | 1.5 |
| Scenario B: End-loaded incentives | Incentives concentrated at later milestones (minimal early compensation) | 10 | 10 | 160 | 2 | 5 | 2.5 | 1.5 | 1.5 |
| Scenario C: High staff effort | Flat incentives with substantially higher staff effort per stage (e.g., qualitative/high-touch study) | 60 | 60 | 60 | 2 | 5 | 30 | 60 | 60 |

1M: 1-month; 3M: 3-month.
